# Cortical white matter microstructural alterations underlying the impaired gamma-band auditory steady-state response in schizophrenia

**DOI:** 10.1038/s41537-024-00454-4

**Published:** 2024-03-12

**Authors:** Daisuke Koshiyama, Ryoichi Nishimura, Kaori Usui, Mao Fujioka, Mariko Tada, Kenji Kirihara, Tsuyoshi Araki, Shintaro Kawakami, Naohiro Okada, Shinsuke Koike, Hidenori Yamasue, Osamu Abe, Kiyoto Kasai

**Affiliations:** 1 Department of Neuropsychiatry, Graduate School of Medicine, The University of Tokyo, Tokyo, Japan; 2 Department of Community Mental Health and Law, National Institute of Mental Health, National Center of Neurology and Psychiatry, Tokyo, Japan; 3 The International Research Center for Neurointelligence (WPI-IRCN) at Institutes for Advanced Study (UTIAS), The University of Tokyo, Tokyo, Japan; 4 Disablity Services Office, The University of Tokyo, Tokyo, Japan; 5 Department of Psychiatry, Teikyo University Hospital, Kawasaki, Japan; 6 University of Tokyo Institute for Diversity & Adaptation of Human Mind (UTIDAHM), Tokyo, Japan; 7 Center for Evolutionary Cognitive Sciences, Graduate School of Art and Sciences, The University of Tokyo, Tokyo, Japan; 8 Department of Psychiatry, Hamamatsu University School of Medicine, Hamamatsu, Japan; 9 Department of Radiology, Graduate School of Medicine, The University of Tokyo, Tokyo, Japan

**Keywords:** Neural circuits, Schizophrenia

## Abstract

The gamma-band auditory steady-state response (ASSR), primarily generated from the auditory cortex, has received substantial attention as a potential brain marker indicating the pathophysiology of schizophrenia. Previous studies have shown reduced gamma-band ASSR in patients with schizophrenia and demonstrated correlations with impaired neurocognition and psychosocial functioning. Recent studies in clinical and healthy populations have suggested that the neural substrates of reduced gamma-band ASSR may be distributed throughout the cortices surrounding the auditory cortex, especially in the right hemisphere. This study aimed to investigate associations between the gamma-band ASSR and white matter alterations in the bundles broadly connecting the right frontal, parietal and occipital cortices to clarify the networks underlying reduced gamma-band ASSR in patients with schizophrenia. We measured the 40 Hz ASSR using electroencephalography and diffusion tensor imaging in 42 patients with schizophrenia and 22 healthy comparison subjects. The results showed that the gamma-band ASSR was positively correlated with fractional anisotropy (an index of white matter integrity) in the regions connecting the right frontal, parietal and occipital cortices in healthy subjects (*β* = 0.41, corrected *p* = 0.075, uncorrected *p* = 0.038) but not in patients with schizophrenia (*β* = 0.17, corrected *p* = 0.46, uncorrected *p* = 0.23). These findings support our hypothesis that the generation of gamma-band ASSR is supported by white matter bundles that broadly connect the cortices and that these relationships may be disrupted in schizophrenia. Our study may help characterize and interpret reduced gamma-band ASSR as a useful brain marker of schizophrenia.

## Introduction

The gamma-band auditory steady-state response (ASSR) has received substantial attention as a potential biomarker indicating the pathophysiology of schizophrenia^1–3^. Previous studies have found selectively reduced power and synchronization in response to 40-Hz auditory stimulation in patients with schizophrenia using electroencephalography (EEG)^4–7^ and magnetoencephalography (MEG)^8,9^. A meta-analysis confirmed that the gamma-band ASSR is a robust index of gamma synchronization deficits in patients with schizophrenia^10^. Furthermore, previous studies found correlations of the gamma-band ASSR with cognitive^4,7^ and psychosocial functioning^11,12^ in patients with schizophrenia. Our recent study showed hierarchical pathways from the gamma-band ASSR to psychosocial functioning through neurocognition and clinical symptoms in patients with schizophrenia using structural equation modeling^13^. Investigation of the neural networks underlying the reduction in gamma-band ASSR in patients with schizophrenia may provide useful information for research on the pathophysiology of schizophrenia.

The results from human and animal model studies investigating gamma-band ASSR generation suggest that γ-aminobutyric acid (GABA)-ergic interneurons and glutamatergic neurons are of central importance, particularly cortical parvalbumin-positive GABA-ergic interneurons and pyramidal neurons^14,15^. As postmortem studies of the brains of patients with schizophrenia have demonstrated reduced expression of the GABA-synthesizing enzyme glutamic acid decarboxylase 67 (GAD67)^16^ and parvalbumin^17^, this suggests it may be a mechanistic underpinning of gamma-band ASSR dysfunction. Indeed, gamma oscillations in response to steady-state stimulation are associated with GABAergic interneuron dysfunction in schizophrenia^18,19^. Furthermore, studies using animal model relevant to schizophrenia have also indicated that altered N-methyl-D-aspartate (NMDA) receptor signaling onto parvalbumin-positive interneurons results in gamma oscillation deficits in cortical microcircuits^20,21^.

While cellular studies of the gamma-band ASSR have indicated prominent roles of GABAergic and glutamatergic neurons, the macro organization/coordination of these microcircuits across brain regions to produce the gamma-band ASSR has not been comprehensively characterized. Previous EEG studies using current source density estimation have demonstrated that the gamma-band ASSR is generated by estimated sources concentrated in the frontal and temporal regions in the left hemisphere and sources distributed across frontal, temporal, parietal and occipital brain regions in the right hemisphere^22–25^. A magnetoencephalography (MEG)-magnetic resonance imaging (MRI) study also showed positive correlations between the gamma-band ASSR and cortical thickness in the temporal and frontal lobes, especially in the bilateral auditory and premotor cortices^26^. Electrocorticography (ECoG) in patients undergoing neurosurgical interventions revealed prominent gamma oscillations in response to 40-Hz auditory stimulation in regions concentrated in the frontal and temporal regions in the left hemisphere and regions distributed across the frontal, temporal and parietal cortices in the right hemisphere^27^.

To further characterize the neural substrates of reduced gamma-band ASSR in patients with schizophrenia, we recently applied novel analytic methods to assess the connectivity among these estimated sources using Granger causality analysis and EEG data from patients with schizophrenia and healthy comparison subjects^25^. This method revealed that the connectivity between the frontal sources and occipital sources underlying the gamma-band ASSR was reduced in patients with schizophrenia. While previous studies have reported reduced gamma-band ASSR primarily in the auditory cortex in patients with schizophrenia^6,23,24^, recent studies with schizophrenia cohorts and healthy cohorts have suggest that the neural substrates of reduced gamma-band ASSR may be more widespread in the cortices, including the auditory cortex, especially in the right hemisphere in patients with schizophrenia^22–27^.

According to previous diffusion tensor imaging (DTI) studies, patients with schizophrenia show severe white matter microstructural alterations across the whole brain^28–32^. Patients with schizophrenia show more severe white matter microstructural alterations in the bundles connecting a wide range of brain regions, such as the corpus callosum, superior longitudinal fasciculus, and superior fronto-occipital fasciculus, than healthy subjects and patients with bipolar disorder, autism spectrum disorder or major depressive disorder^29^. Given these previous findings^28–31^ and the distributed source locations of the gamma-band ASSR in the right hemisphere^22–24,27^, investigating the associations of white matter alterations in the superior fronto-occipital fasciculus with the gamma-band ASSR may provide important information on the pathophysiology of schizophrenia. Despite its name, the superior fronto-occipital fasciculus does not merely connect the frontal and occipital regions; a large portion of the superior fronto-occipital fasciculus also connects to the parietal region^33^.

This study aimed to investigate associations of white matter alterations in the right superior fronto-occipital fasciculus with the gamma-band ASSR to clarify the networks underlying the gamma-band ASSR. First, we attempted to replicate previous findings regarding gamma-band ASSR reduction and white matter microstructural alterations in the fronto-occipital fasciculus in patients with schizophrenia compared to healthy subjects. Second, we performed correlation analyses of the relationship between white matter microstructural alterations in the fronto-occipital fasciculus and gamma-band ASSR impairment in patients with schizophrenia and healthy comparison subjects.

## Materials and Methods

### Participants

EEG and DTI data were obtained from 42 patients with schizophrenia and 22 healthy comparison subjects. Participants had also participated in our previous gamma-band ASSR studies^11,34,35^ and a DTI study^29–31^. Patients were recruited from outpatient and inpatient units at the University of Tokyo Hospital. The healthy comparison group was recruited through advertisements at several universities in Tokyo. Patients were diagnosed using the Diagnostic and Statistical Manual of Mental Disorders, fourth edition (DSM-IV). Healthy comparison subjects did not have a personal history of psychiatric disease or a family history of Axis I disorders in first-degree relatives. Exclusion criteria for both groups were as follows: neurological illness, traumatic brain injury with loss of consciousness lasting more than five minutes, history of electroconvulsive therapy, low premorbid intelligence quotient (IQ < 70), previous alcohol/substance abuse or addiction, or a hearing impairment (assessed with a hearing test in both ears at a sound pressure level of 30 dB and a tone of 1000 Hz as well as 40-dB at 4000 Hz by audiometer). Written informed consent was obtained from each subject before participation. The Research Ethics Committee of the Faculty of Medicine at the University of Tokyo approved this study (Approval nos. 629, 2226 and 3150).

The Brief Psychiatric Rating Scale (BPRS)^36^ and modified Global Assessment of Functioning (GAF)^37,38^ were used to assess global clinical symptoms and functioning in patients with schizophrenia. Forty patients with schizophrenia took antipsychotic medication. The dose of antipsychotics was converted to an equivalent dose of chlorpromazine^39^.

### EEG measurement and analyses

Two EEG measurement systems were used in this study. A 64-channel Geodesic EEG System (Electrical Geodesics Inc., Eugene, OR) was used to obtain EEG data using a GSN 200 cap from 24 patients with schizophrenia and 18 healthy comparison subjects and a HydroCel GSN 130 cap from 14 patients with schizophrenia and 4 healthy comparison subjects. Electrodes were referenced to the vertex, and impedances were maintained below 50 kΩ. The sampling rate was 250 Hz. The analog filter bandpass was set at 0.1–100 Hz. EEG data on the frontocentral electrode site (FCz) were analyzed. EEG data were analyzed by using EEGLAB^40^. The continuous EEG data were re-referenced to an average reference, a high-pass filter (1 Hz) and a notch filter (50 Hz) were applied to them to remove artifacts, and they were segmented from −250 to 750 ms relative to the stimulus onset. Independent component analysis was used for eye blink correction, and epochs exceeding ±100 μV at any electrode were rejected.

An EEG acquisition system with active electrodes (Polymate II, AP2516; Miyuki Giken, Tokyo, Japan), which is compact with a maximum of 16 channels, was used to obtain EEG data from 4 patients with schizophrenia. The EEG data were acquired at Fz and Cz and referenced to the left mastoid. A ground electrode was located at the right mastoid. Vertical electrooculography data were recorded from electrodes above and below the right eye. The sampling rate was set at 1000 Hz with the analog filter bandpass set at 0.05–333 Hz. All electrode impedances were below 50 kΩ. EEG data were preprocessed using Vision Analyzer (version 2.1, Brain Products, Munich, Germany). EEG data from the frontal electrode site (Fz) were analyzed. Epochs were extracted from –250 to 750 msec. Eyeblink artefacts were corrected from the Gratton & Coles method^41^. We excluded epochs exceeding ±75 µV.

The ASSR paradigm used in this study has been described in detail elsewhere^11,34,35^ and is similar to paradigms used in studies from different laboratories^12,13,23–25,42,43^. Briefly, subjects were instructed to relax with their eyes open, and they received auditory stimuli presented binaurally through inserted earphones. Click sounds (80 dB, 1 ms) presented in 500 ms trains at 40 Hz served as the auditory stimuli. Click sound trains were 200 trains. The intertrain interval was 500 ms.

Time–frequency analyses with the short-term Fourier transform were performed using the timefreq() function of EEGLAB (https://github.com/sccn/eeglab/blob/develop/functions/timefreqfunc/timefreq.m). The intertrial phase coherence (ITC) and event-related spectral perturbation (ERSP) were calculated as indices of the ASSR. The ITC indicates phase consistency across trials and ranges between 0 (random phase across trials) and 1 (identical phase across trials). The ERSP indicates event-related changes in power relative to a prestimulus baseline. Decreases in the ITC and/or ERSP reflects reduced neural responses to auditory steady-state stimulation. The mean ITC or ERSP was calculated by averaging the data over stimulation time (0–500 ms) and stimulation frequency (40 Hz: 36–45 Hz). The ITC or ERSP were not significantly different among the three methods of EEG measurement (a 64-channel Geodesic EEG System using GSN 200 cap (*N* = 42) and HydroCel GSN 130 cap (*N* = 18) and an EEG acquisition system with active electrodes (*N* = 4); ITC, *F*_2,61_ = 2.0, *p* = 0.14; ERSP, *F*_2,61_ = 0.87, *p* = 0.42).

### DTI measurement and analyses

Three DTI protocols were used in this study. Protocol A was used to obtain DTI data from 14 patients with schizophrenia and 6 healthy comparison subjects, Protocol B was used for 5 patients with schizophrenia, and Protocol C was used for 23 patients with schizophrenia and 16 healthy comparison subjects.

In Protocol A, whole-brain axial DTI scanning was performed on a 3.0 T GE Signa scanner using an eight-channel brain coil with the following parameters: two-dimensional diffusion-weighted spin‒echo EPI sequence, TR = 20 s, TE = 55.3 ms, acquisition matrix = 128 × 128, reconstruction matrix = 256 × 256, ASSET acceleration factor = 2, FOV = 240 × 240 mm, slice thickness = 2.4 mm, voxel size = 0.938 × 0.938 × 2.4 mm, and number of slices = 67. A diffusion sensitization gradient was applied with 30 noncollinear gradient directions and b values of 0 and 1000 s/mm^2^.

In Protocol B, whole-brain axial DTI scanning was performed on a 3.0 T GE Discovery MR750W scanner using a 32-channel brain coil with the following parameters: two-dimensional diffusion-weighted spin‒echo EPI sequence, TR = 16 s, TE = 95.7 ms, acquisition matrix = 128 × 128, reconstruction matrix = 256 × 256, ASSET acceleration factor = 2, FOV = 256 × 256 mm, slice thickness = 2.5 mm, voxel size = 1 × 1 × 2.5 mm, and number of slices = 64. A diffusion sensitization gradient was applied with 30 noncollinear gradient directions and b values of 0 and 1000 s/mm^2^.

In Protocol C, whole-brain axial DTI scanning was performed on a 3.0 T GE Discovery 750w scanner using a 24-channel brain coil with the following parameters: two-dimensional diffusion-weighted spin‒echo EPI sequence, TR = 13 s, TE = 86.1 ms, acquisition matrix = 96 × 96, reconstruction matrix = 128 × 128, ASSET acceleration factor = 2, FOV = 240 × 240 mm, slice thickness = 2.5 mm, voxel size = 1.875 × 1.875 × 2.5 mm, and number of slices = 60. A diffusion sensitization gradient was applied with 30 noncollinear gradient directions and b values of 0, 1000, 1500 and 2000 s/mm^2^.

Quality control included visual inspection of the original T1-weighted images by two independent MRI researchers and exclusion of images with any abnormal findings (for example, large cerebellar cysts and cavum septum pellucidi), checking of the scan parameters for each DTI scan and the exclusion of DTI data obtained with incorrect parameters, and exclusion of DTI data that failed processing with FSL 5.0 (https://fsl.fmrib.ox.ac.uk/fsl) tract-based spatial statistics (TBSS).

DTI image processing steps included head motion and eddy current correction using eddy_correct (FSL 5.0). We employed fractional anisotropy (FA) as a DTI index. We also employed mean diffusivity (MD), axial diffusivity (AD), and radial diffusivity (RD) as DTI indices in the post hoc analyses. Estimation of the DTI indices was performed using dti_fit (FSL 5.0). TBSS, using the ENIGMA-DTI template and JHU ROIs, was applied to extract local values of the DTI indices based on ENIGMA-DTI protocols (http://enigma.ini.usc.edu/protocols/dti-protocols/). FA is thought to indicate white matter integrity and the underlying characteristics of white matter microstructure, such as the directionality, diameter, and density of axonal fibers as well as myelin sheath thickness. FA is derived from the degree of anisotropy of the following eigenvalues of the diffusion tensor: λ_1_, λ_2_, and λ_3_. The largest eigenvalue (λ_1_), i.e., the AD, is a possible marker for axonal injury. The average of the two smaller eigenvalues λ_2_ and λ_3_, i.e., the RD, is considered an indicator of myelin damage. The MD is the average of all three eigenvalues^44,45^. Finally, we obtained the DTI indices (FA, MD, AD, and RD) of the superior fronto-occipital fasciculus^28^. As the FA of the superior fronto-occipital fasciculus significantly differed among the three protocols (Protocol A, *N* = 25; Protocol B, *N* = 5; Protocol C, *N* = 39; *F*_2, 61_ = 3.4, *p* = 0.04), protocol was included as a covariate in the following comparison analyses, and *z* scores of DTI indices were used in the following correlation analyses.

### Statistical analyses

SPSS (version 28.0.1.0, IBM Corp., Armonk, NY, USA) was used for all statistical analyses. *χ*^*2*^ tests and independent-sample *t* tests were employed for comparison of demographic characteristics between the groups. *t* tests were used for comparison of ITC and ERSP values between the groups (the significance level was set at *p* < 0.05 [one-tailed], as previous studies have shown that the ITC and ERSP are reduced in patients with schizophrenia^4–7,10^). Cohen’s *d* was calculated for the comparison of ITC and ERSP values between groups as effect size measure. ITC was employed in subsequent analyses because ITC was significantly reduced in patients with schizophrenia while ERSP was not.

Comparison of patients and healthy comparison subjects in terms of the FA of the right superior fronto-occipital fasciculus was performed using regression analysis using age, sex and DTI protocol as covariates (the significance level was set at *p* < 0.05 [one-tailed] since previous studies have shown that the FA of the region is impaired in patients with schizophrenia^28,29^). When the difference in the FA of the right superior fronto-occipital fasciculus between the groups was significant, post hoc comparison analyses were performed for DTI indices (MD, AD and RD) of the right superior fronto-occipital fasciculus. Standardized *β* was calculated for comparison of DTI indices between groups as an effect size measure.

Regression analysis using age and sex as covariates was performed to examine the association between ITC values and *z* scores of the FA of the right superior fronto-occipital fasciculus in each group (the significance level was set at *p* < 0.05 [two-tailed]). Multiple comparisons of the two groups of patients with schizophrenia and healthy comparison subjects were addressed using Bonferroni correction. When the association was significant, post hoc regression analyses were performed for associations between ITC values and *z* scores of DTI indices (MD, AD and RD) of the right superior fronto-occipital fasciculus.

As supplementary analyses for the specificity of our hypothesis, we performed correlation analysis of the relationship between ITC values and the FA of the left fronto-occipital fasciculus and that between ITC values and the FA of the fornix as comparison white matter bundles, which may unlikely contribute as a main generator of the gamma-band ASSR.

## Results

### The gamma-band ASSR

**Fig. 1:**
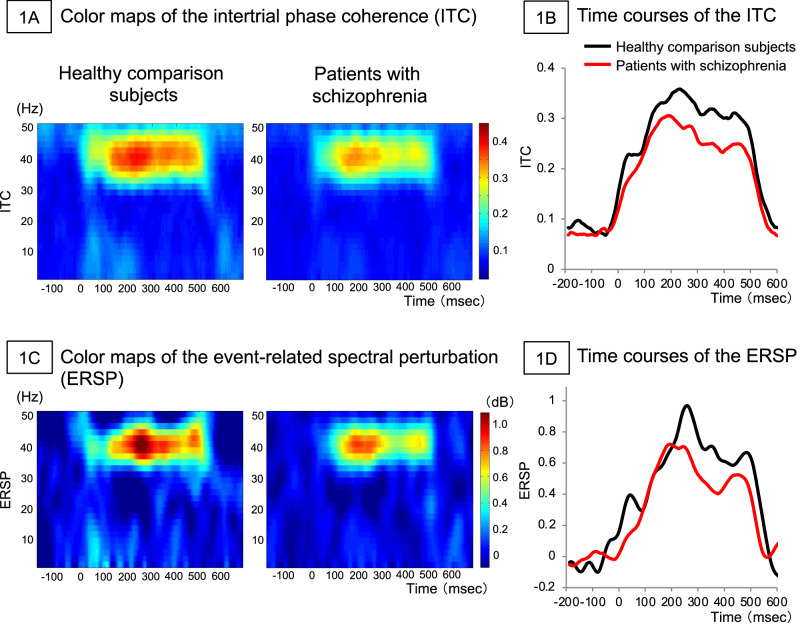
Color maps and time courses of the gamma-band auditory steady-state response (ASSR). The figures show color maps (**A**) and time courses (**B**) of the intertrial phase coherence (ITC) and color maps (**C**) and time courses (**D**) of the event-related spectral perturbation (ERSP).

The demographic characteristics of the subjects are shown in Table 1. The grand average time-frequency maps for the ITC and ERSP, which are indices of the ASSR, are shown in Fig. 1A and C. The time courses of the ITC and ERSP are shown in Fig. 1B and D. The ITC was significantly different between patients with schizophrenia and healthy comparison subjects (*t*_62_ = 1.7, *p* = 0.049, *d* = 0.44). The effect size was similar to that of the comparison between patients with schizophrenia and healthy comparison subjects in the previous meta-analysis conducted by Thuné et al*.*^10^ The evoked power was not significantly different between patients with schizophrenia and healthy comparison subjects (*t*_62_ = 1.1, *p* = 0.14, *d* = 0.29). Therefore, in subsequent analyses, ITC was employed.

**Table 1:** The demographic characteristics of healthy comparison subjects and patients with schizophrenia.

	Healthy comparison subjects	Patients with schizophrenia	Statistics
N (sex ratio M/F)^a^	22 (7/15)	42 (27/15)	*χ*^2^ = 6.1, *df* = 1, *p* = 0.01^*^
Age (years)^b^	30.9 (7.4)	27.9 (8.8)	*t*_62_ = 1.4, *p* = 0.18
Duration of illness (years)^c^		5.9 (7.3)	
BPRS score^d^		40.5 (11.1)	
GAF score^c^		41.4 (11.6)	
Antipsychotic dose (mg/day)		576.5 (485.3)	

All values are shown as means (standard deviations); Age is at diffusion tensor imaging measurement; *Statistical significance set at *p* < 0.05; ^a^Chi-square test; ^b^Independent *t*-test. ^c^One patient had no data. ^d^Two patients had no data.

*BPRS* Brief Psychiatric Rating Scale, *GAF* Global Assessment of Functioning.

### DTI index of the right superior fronto-occipital fasciculus

**Fig. 2:**
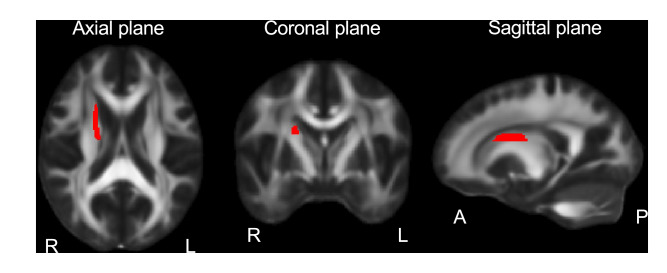
Regions of the superior fronto-occipital fasciculus. The superior fronto-occipital fasciculus is composed of long bidirectional fronto-parietal-occipital projections. A anterior, L left, P posterior, R right.

The FA of the right superior fronto-occipital fasciculus (Fig. 2) was significantly reduced in patients with schizophrenia compared to healthy subjects (*β* = –0.36, *p* = 6.8 × 10^–4^).

The effect size was similar to the effect sizes of the comparison between patients with schizophrenia and healthy subjects in the previous meta-analysis conducted by Kelly et al. (*d* = –0.29)^28^ and our mega-analysis (*d* = –0.36)^29^. Post hoc analyses showed that the DTI indices of the right superior fronto-occipital fasciculus were significantly increased in patients with schizophrenia in terms of RD (*β* = 0.20, *p* = 0.043) but not MD (*β* = 0.16, *p* = 0.072) or AD (*β* = 0.10, *p* = 0.16). Additionally, the FA of the left superior fronto-occipital fasciculus (*β* = –0.42, *p* = 1.2 × 10^–4^) and that of the fornix (*β* = –0.43, *p* = 1.2 × 10^–4^) were also significantly reduced in patients with schizophrenia compared to healthy subjects.

### Associations between the gamma-band ASSR and DTI indices of the right superior fronto-occipital fasciculus

**Fig. 3:**
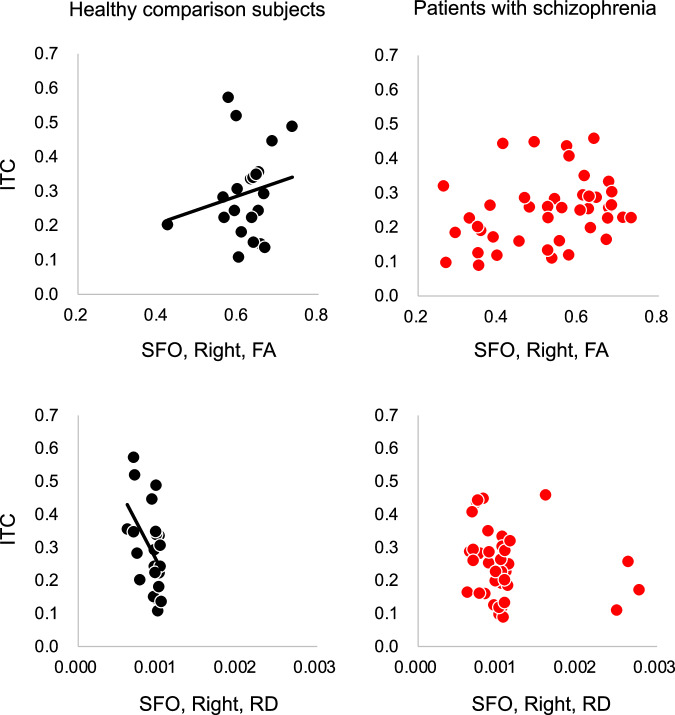
Correlations between the intertrial phase coherence (ITC) of the gamma-band auditory steady-state response (ASSR) and diffusion tensor imaging (DTI) indices of the superior fronto-occipital fasciculus (SFO). The ITC was correlated with the FA of the right SFO in healthy comparison subjects (*β* = 0.41, corrected *p* = 0.075, uncorrected *p* = 0.038) but not in patients with schizophrenia (*β* = 0.17, corrected *p* = 0.46, uncorrected *p* = 0.23). Post hoc analyses revealed that the ITC was associated with the RD of the right SFO in healthy comparison subjects (*β* = –0.45, *p* = 0.025) but not in patients with schizophrenia (*β* = –0.18, *p* = 0.22). DTI indices of the right SFO, corrected for age, sex and DTI protocol, were employed in these scatter plots. FA fractional anisotropy, RD radial diffusivity.

The ITC was significantly associated with the FA of the right superior fronto-occipital fasciculus in healthy subjects (*β* = 0.41, corrected *p* = 0.075, uncorrected *p* = 0.038) but not in patients with schizophrenia (*β* = 0.17, corrected *p* = 0.46, uncorrected *p* = 0.23; Fig. 3) according to the uncorrected *p* value for multiple comparisons. The post hoc analyses showed that the ITC was significantly associated with the RD of the right superior fronto-occipital fasciculus in healthy subjects (*β* = –0.45, *p* = 0.025) but not in patients with schizophrenia (*β* = –0.18, *p* = 0.22); the ITC was not significantly associated with the MD or AD of the right superior fronto-occipital fasciculus in healthy comparison subjects (MD, *β* = –0.19, *p* = 0.44; AD, *β* = 0.37, *p* = 0.11) or in patients with schizophrenia (MD, *β* = –0.17, *p* = 0.25; AD, *β* = –0.15, *p* = 0.32).

Additionally, the ITC was not significantly correlated with the FA of the left superior fronto-occipital fasciculus or that of the fornix in healthy subjects (left superior fronto-occipital fasciculus, *β* = 0.07, *p* = 0.73; fornix, *β* = 0.10, *p* = 0.63) or in patients with schizophrenia (left superior fronto-occipital fasciculus, *β* = 0.11, *p* = 0.45; fornix, *β* = 0.06, *p* = 0.67).

The antipsychotic dose was significantly correlated with the ITC (*r* = –0.38, *p* = 0.012), the FA of the right superior fronto-occipital fasciculus (*r* = –0.34, *p* = 0.026), and the RD of the right superior fronto-occipital fasciculus (*r* = 0.36, *p* = 0.019) in patients with schizophrenia. Therefore, we performed correlation analyses between the ITC and the FA (*β* = 0.14, *p* = 0.34) and RD (*β* = –0.14, *p* = 0.33) of the right superior fronto-occipital fasciculus considering the antipsychotic dose; however, the results were not different. The antipsychotic dose was significantly correlated with the BPRS score (*r* = 0.43, *p* = 6.0 × 10^−3^). The BPRS scores were also significantly correlated with the ITC (*r* = –0.43, *p* = 5.0 × 10^−3^), the FA of the right superior fronto-occipital fasciculus (*r* = –0.41, *p* = 9.1 × 10^−3^), and the RD of the right superior fronto-occipital fasciculus (*r* = 0.60, *p* = 4.5 × 10^−5^) in patients with schizophrenia. Partial correlational analyses controlling for the antipsychotic dose showed that BPRS scores were significantly correlated with the RD (*r* = 0.53, *p* = 4.8 × 10^−4^) of the right superior fronto-occipital fasciculus and were correlated with ITC (*r* = –0.31, *p* = 0.053) and the FA (*r* = –0.31, *p* = 0.053) of the right superior fronto-occipital fasciculus at the trend level in patients with schizophrenia. Therefore, the correlation of antipsychotic dose with ITC, FA, and RD is thought to reflect the associations of clinical symptoms with gamma-band ASSR and white matter microstructural alterations in patients with schizophrenia rather than the medication effect.

For supplementary information, we examined the relationship between the evoked power and the FA of the right superior fronto-occipital fasciculus. However, the evoked power was not significantly associated with the FA of the right superior fronto-occipital fasciculus in patients with schizophrenia (*β* = –0.05, *p* = 0.76) or in healthy comparison subjects (*β* = 0.22, *p* = 0.24) according to regression analyses using age and sex as covariates. Furthermore, we showed the results of 47 electrodes that are shared between the GSN 200 and HydroCel GSN 130 caps from a portion of the samples, other than the data from Polymate II, in Supplementary Table.

## Discussion

The results of our study showed that white matter integrity in the regions connecting the right frontal, parietal and occipital cortices was positively correlated with the gamma-band ASSR in healthy subjects but not in patients with schizophrenia according to the uncorrected *p* value for multiple comparisons. These results imply that white matter microstructural alterations in the bundle underlie the impaired gamma-band ASSR in patients with schizophrenia. The ITC, which is an index of the gamma-band ASSR, was significantly reduced in patients with schizophrenia in this study. The FA of the right superior fronto-occipital fasciculus was significantly reduced in patients with schizophrenia. Post hoc analyses revealed that the DTI indices of the right superior fronto-occipital fasciculus were significantly increased in patients with schizophrenia in terms of RD but not MD or AD. Furthermore, the ITC was significantly positively correlated with the FA of the right superior fronto-occipital fasciculus in healthy subjects according to the uncorrected *p*-value for multiple comparisons, but this correlation was not observed in patients. Additionally, post hoc analyses showed that the ITC was significantly negatively correlated with the RD of the right superior fronto-occipital fasciculus in healthy subjects but not in patients. Moreover, the ITC was not significantly correlated with the FA of the left superior fronto-occipital fasciculus or that of the fornix in either group.

The results of this study imply that structure‒function relationships that are functional in healthy comparison subjects may be impaired in patients with schizophrenia. At the microscopic level, the RD is considered an indicator of myelin sheath integrity^44,45^. Furthermore, the gamma-band ASSR is considered to reflect parvalbumin-positive GABAergic interneuron function^14,15^ and is reduced in patients with schizophrenia, reflecting dysfunction of GABAergic interneurons^18,19^. Therefore, we speculated that dysfunction of the fronto-occipital fasciculus due to myelin damage to GABAergic interneurons may reduce the gamma-band ASSR in patients with schizophrenia. At the macro level, our previous EEG connectivity study reported that the connectivity between the right frontal and occipital regions underlying the gamma-band ASSR is impaired in patients with schizophrenia^25^. An electrocorticography study conducted by Tada et al. showed that sources of the gamma-band ASSR were distributed throughout the right frontal and parietal regions in humans^27^. The current results provide more precise spatial information on the microstructural alterations underlying reduced gamma-band ASSR in patients with schizophrenia. The fronto-occipital fasciculus may subserve not only sensory processing but also higher-order neurocognition; thus, our findings may provide important information regarding the use of the gamma-band ASSR in translational research on schizophrenia.

There are several limitations of this study. First, we employed two EEG systems and three DTI protocols in this study. Although we confirmed that there were no differences in EEG data among the systems and employed *z* scores of DTI indices because of the significant differences in DTI indices among the protocols, the influence of these differences on the results cannot be completely ruled out. Second, we did not employ source estimation for EEG signals. Source-based or functional connectivity analyses have been performed in several previous studies, suggesting that patients with schizophrenia exhibit alterations in multiple neural networks underlying the ASSR^23–25^. In our previous study, dividing scalp EEG data into estimated sources was shown to reduce group differences in gamma-band ASSR between patients with schizophrenia and healthy subjects^24^. Therefore, in the present study, we focused on the most prominent electrode, FCz, according to the topographies of the 64-channel EEG (Supplementary Figure). Future studies using source estimation analyses in a large cohort of samples will be able to disentangle complicated associations and clarify more detailed associations between the gamma-band ASSR and white matter microstructure in patients with schizophrenia. Third, the correlation between the ITC of the gamma-band ASSR and the FA of the right superior fronto-occipital fasciculus in healthy subjects was significant at the uncorrected *p* value but not significant at the corrected *p* value for multiple comparisons. We used ITC but not ERSP because only ITC was significantly reduced in patients with schizophrenia in this study. However, several other studies have shown a decrease in the power of the ASSR in patients with schizophrenia^10^. We used the FA of the right superior fronto-occipital fasciculus, while several other studies have shown alterations in white matter in other regions in patients with schizophrenia^28,29^. Therefore, future studies with larger samples including both the phase and power of ASSR and multiple regions of white matter are necessary for clarifying white matter alterations underlying reduced gamma-band ASSR in patients with schizophrenia.

In conclusion, the current study showed that the gamma-band ASSR was supported by white matter bundles that broadly connect the cortices, and these relationships may be impaired in schizophrenia. The possibility that the decreased ASSR may result not only from abnormalities in the sensory cortex but also from network disruption in the association cortex may provide important information for future translational studies on schizophrenia using the ASSR as a brain marker to elucidate the pathophysiology of the disorder and its relationship to functional outcomes.

### Supplementary information

Supplementary Table

Supplementary Figure

## Data Availability

The data may be provided upon reasonable request.
