# Supplementary material for: Cortical white matter microstructural alterations underlying the impaired gamma-band auditory steady-state response in schizophrenia
Source: Schizophrenia (Heidelb). 2024 Mar 12;10(1):32. doi: 10.1038/s41537-024-00454-4 (PMC10933284; doi:10.1038/s41537-024-00454-4)
Supplement: Supplementary file 2 — Supplementary Figure [file 41537_2024_454_MOESM2_ESM.docx]

**Supplementary Figure** Topographies of intertrial phase coherence and event-related spectral perturbation at FCz in each group

Legend: Circles indicate FCz.
